# Collagen Membrane Fixation Technique Affects Its Space Maintenance Capacity After Flap Closure: An Ex Vivo Analysis

**DOI:** 10.1111/clr.70146

**Published:** 2026-06-12

**Authors:** Muhammad H. A. Saleh, Javier Calatrava, Nataly Zambrana, Lucrezia Parma‐Benfenati, Hamoun Sabri, Istvan Urban, Hom‐Lay Wang

**Affiliations:** ^1^ Department of Periodontics and Oral Medicine, School of Dentistry University of Michigan Ann Arbor Michigan USA; ^2^ Section of Graduate Periodontology, Faculty of Odontology University Complutense Madrid Spain; ^3^ Department of Oral Medicine, Infection Control and Immunity Harvard University Boston Massachusetts USA; ^4^ Department of Prosthetic Dentistry University of Szeged Szeged Hungary; ^5^ Urban Regeneration Institute Budapest Hungary

**Keywords:** alveolar ridge augmentation, bone regeneration, cadaver, cone‐beam computed tomography

## Abstract

**Aims:**

To quantify immediate graft displacement following tension‐free flap closure in non‐contained alveolar ridge defects regenerated using three membrane fixation strategies in an ex vivo human model.

**Methods:**

Eighteen sites from six fresh cadaveric heads with non‐contained defects underwent horizontal guided bone regeneration using deproteinized bovine bone mineral and a collagen membrane stabilized by no fixation (NF), periosteal sutures (PS), or titanium fixation pins (FP). Techniques were applied sequentially. Cone‐beam CT scans were obtained at baseline, post‐grafting, and after passive closure. Linear ridge width was measured at 12 standardized positions, and graft volumes were segmented from STL reconstructions. Mixed‐effects multilevel linear regression assessed width changes, while Friedman and paired Wilcoxon tests evaluated volumetric outcomes, with Tukey‐adjusted contrasts for intergroup comparisons.

**Results:**

Fixation pins virtually eliminated graft displacement (−6.7 ± 87 mm^3^) and achieved the highest stability (81.2% ± 8.2%), significantly outperforming periosteal sutures (−96 ± 165 mm^3^; 70.1% ± 4.5%) and no fixation (−247 ± 150 mm^3^; 44.4% ± 17.9%) (loss *p* = 0.030; stability *p* = 0.002). Ridge collapse was greatest with no fixation (−1.37 ± 0.85 mm) compared with PS (−0.40 ± 0.62 mm) and FP (−0.28 ± 0.61 mm). After adjustment, PS and FP reduced collapse by 0.96 mm (*p* = 0.012) and 1.11 mm (*p* = 0.004), respectively.

**Conclusion:**

Even after passive flap release and tension‐free closure, membrane fixation, particularly with titanium pins, provides superior resistance to graft displacement in non‐contained defects, while unfixed membranes allow substantial graft loss and are therefore not recommended in non‐contained defects.

## 1 Introduction

Implant therapy often necessitates either staged or simultaneous bone augmentation due to post‐extraction alveolar bone changes (Schropp et al. 2003; Araújo and Lindhe 2009). This physiological remodeling process can make implant placement especially challenging in the posterior mandible with severe alveolar ridge atrophy (Elnayef et al. 2017; Urban et al. 2017). When planning implant‐supported restorations, alveolar ridge augmentation (ARA) techniques, particularly guided bone regeneration (GBR) procedures, are often necessary to increase ridge volume in order to place dental implants (Rocchietta et al. 2008; Urban and Monje 2019).

In 2006, Wang and Boyapati described four major biological principles necessary for predictable bone regeneration (PASS principles). The space maintenance principle is of the utmost importance to facilitate adequate space for bone regeneration (Wang and Boyapati 2006). The barrier function for selective cell exclusion is another key element in GBR (Lim et al. 2018). Several studies have reported on the effectiveness of GBR utilizing a combination of absorbable collagen membranes and bone graft materials (Merli et al. 2014; Lee et al. 2022; Leong et al. 2015; Cardaropoli et al. 2013; Llambés et al. 2007; Urban, Lozada, et al. 2013; Urban, Nagursky, et al. 2013).

Even though absorbable barrier membranes were associated with a weighted mean complication rate of 18.3%, most of these complications were related to the soft tissue management, which can have a direct impact on the success of the bone regeneration (Lim et al. 2018). In line with these principles, a recent review by Urban et al. (2023) has highlighted the importance of graft and membrane stability for achieving predictable vertical and horizontal ridge augmentation, particularly in posterior atrophic ridges. One of the more widely adopted approaches employs a resorbable collagen membrane secured with titanium pins (Urban, Lozada, et al. 2013; Urban, Nagursky, et al. 2013). In this technique, membrane fixation seemed to increase the osteogenic potential of the graft materials during GBR (An et al. 2022; Wessing et al. 2018).

Previous in vitro studies have reported that wound closure can induce displacement of the bone substitute, leading to partial collapse of the collagen membrane at the coronal portion of the augmented site in contained defects (Mir‐Mari et al. 2017, 2016). More recently, ex vivo investigations have expanded this concept to clinically relevant flap advancement scenarios, demonstrating that both periosteal sutures and fixation pins influence graft displacement during primary wound closure (Raabe, Cafferata, Zhou, et al. 2025; Zhou et al. 2025). Although membrane fixation has been consistently associated with improved graft and membrane stability in guided bone regeneration (Urban et al. 2017, 2016; An et al. 2022; Mizraji et al. 2023; Wang et al. 2022), it is often regarded as one of the most technically demanding steps of the procedure (An et al. 2022). This complexity is largely related to technique sensitivity and anatomical constraints, particularly in lingual sites, as well as the risk of intraoperative complications such as damage to adjacent roots or vital anatomical structures (Urban and Monje 2019; Siar et al. 2011). To address these issues, a periosteal mattress suture using absorbable materials has been proposed as a method to stabilize bone grafts and resorbable membranes. This approach aims to simplify the procedure and reduce the risk of damaging adjacent vital structures during the insertion of pins (tacks) or screws (Urban et al. 2016). Either horizontal periosteal sutures as described by Urban et al. (2016), or continuous periosteal strapping sutures as described by Shalev et al. (2017), or “Lasso sutures” as described by Neiva et al. (2022) can be utilized to secure membranes, thereby minimizing risks and comorbidities associated with buccal ridge augmentation techniques (Shalev et al. 2017). Regardless of the variation in technique, these suture techniques have some limitations, which could affect the outcomes of GBR, such as the potential migration of the particulate graft material in an apico‐coronal direction after flap closure, and the challenge of ensuring stability of grafts in large defects (Mir‐Mari et al. 2017, 2016; Urban et al. 2016).

Although isolating the effect of flap closure on graft displacement in human patients is technically feasible, it is ethically and practically unjustifiable, as it would require additional immediate pre‐ and post‐closure CBCT imaging (Mir‐Mari et al. 2017). Moreover, the biomechanical properties of human periosteal and soft tissues differ fundamentally from those of in vitro or animal models, limiting the generalizability of preclinical findings (Mir‐Mari et al. 2016). To address these gaps, we designed an ex vivo study comparing the immediate displacement of graft particles after flap closure without tension in non‐contained alveolar defects under different fixation strategies (no fixation, periosteal sutures, and fixation pins), thereby determining whether specific fixation techniques can prevent graft material displacement even when optimal flap release has been achieved.

## 2 Materials and Methods

### 2.1 Study Design

This ex vivo human experimental study was conducted to evaluate the dimensional stability of grafted bone particles in GBR procedures using three different membrane fixation techniques. Ethical oversight was obtained from the University of Michigan Institutional Review Board, which granted an exemption.

Each cadaveric sample was utilized for the three surgical techniques:

1. No fixation (NF).
2. Periosteal sutures (PS).
3. Fixation pins (FP).

Linear and volumetric dimensional changes were assessed using cone‐beam computed tomography (CBCT) before and after flap closure.

### 2.2 Study Specimens and Eligibility Criteria

Six fresh, non‐embalmed, partially edentulous human cadaveric heads were obtained through a certified anatomical donation program (University of Michigan Hospital). To preserve intra‐oral structures, specimens were stored at −20°C without formalin. Before use, each head was slowly and evenly thawed by holding it at 2°C for ~48 h, then brought to room temperature (Klop et al. 2017). All measurements were completed within 8 h post‐thaw to avoid autolytic changes such as nuclear vacuolation and eosinophilia, which become pronounced after that interval (Mahalakshmi et al. 2016).

#### 2.2.1 Inclusion Criteria

1. Partially dentate mandibles.
2. Non‐tooth‐bounded alveolar defects.
3. Horizontal defects with remaining bone width ≤ 5 mm.
4. Intact gingival tissues without handling trauma.

#### 2.2.2 Exclusion Criteria

1. Complete edentulism.
2. Contained alveolar defects.
3. Vertical ridge defects.

### 2.3 Study Steps

**FIGURE 1:**
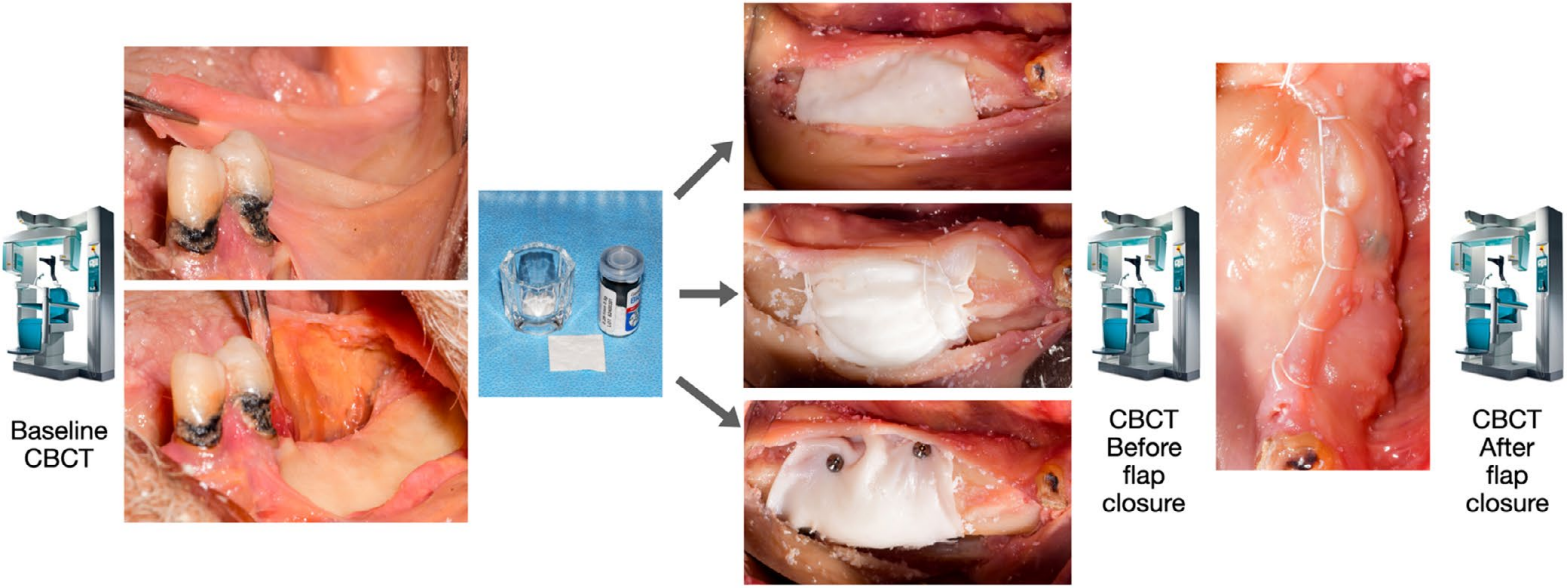
Study design flowchart. Baseline CBCT scans of non‐contained alveolar defects in human cadaver heads were acquired prior to flap opening and tension‐free flap release. GBR was performed sequentially at each site using three fixation strategies: no fixation (top), periosteal sutures (middle), and titanium pins (bottom). CBCT imaging was repeated before and after tension‐free flap closure to quantify linear collapse and volumetric graft displacement for each fixation method.

Each specimen arrived with donor age and sex information, which were recorded first. The experimental steps proceeded in this order to ensure standardization (Figure 1):

- Cone‐beam CT (CBCT) imaging.
- Incision and flap elevation with sufficient flap release, ensuring closure without tension.
- The site received GBR without membrane fixation (NF).
- Cone‐beam CT (CBCT) imaging with the flap being open.
- Tension‐free flap closure.
- Cone‐beam CT (CBCT) imaging after flap closure.
- The same steps were repeated on the same site for the PS, followed by the FP techniques.

### 2.4 Study Materials

All defects were grafted with the same standardized amount of deproteinized bovine bone mineral (DBBM) (Bio‐Oss, Geistlich Pharma, Switzerland; particle size 0.25–1.0 mm, 0.5 g). The full volume of the bone replacement graft (BRG) was placed into the alveolar defect. An absorbable collagen membrane (Bio‐Gide, Geistlich Pharma, Switzerland, 25 × 25 mm) was used to cover each graft. Membranes were pre‐hydrated in sterile saline for 5 min prior to adaptation over the grafted area.

To ensure consistency in defect configuration and baseline volume of biomaterials used, each site underwent the three experimental procedures sequentially. For every application, a fresh vial of bone substitute and a new membrane of identical volume and size were used, the site was then restored to baseline, and the next fixation technique was applied with the same standardized biomaterials.

### 2.5 Experimental Procedure

All procedures were performed by the same surgeon (M.H.A.S.) to ensure procedural consistency across specimens. The most severe horizontal defect of each specimen was employed for the experimental procedure. All surgical steps attempted to simulate the in vivo clinical decision‐making process. A crestal incision was performed on top of the defect, dividing the available keratinized mucosa between both buccal and lingual/palatal flaps. The incision was extended mesio‐distally to the nearest tooth and at least 5 mm beyond the defect in case of a free‐end saddle defect configuration. Mesial buccal vertical releasing incisions were performed, extending beyond the mucogingival junction, to allow for proper defect assessment, flap release, and membrane fixation. Both buccal and lingual flaps were elevated full thickness past the mucogingival junction, 3–4 mm beyond the defect to ensure enough space for membrane fixation with pins, while leaving enough intact periosteum apically for periosteal fixation sutures.

Flap release was performed to allow tension‐free primary intention closure, before the regenerative procedures, in order to standardize flap tension (Figure 1). The buccal flap was released by means of sharp split‐thickness dissection, whereas the lingual flap was released mostly by means of a blunt instrument with the aim of deep separation (Urban et al. 2017, 2024). Then, the three regenerative procedures and membrane fixation techniques were carried out, suturing and unsuturing the flaps between each procedure. Flaps were advanced coronally and sutured by a double‐layer suture, consisting of a first layer of one or two internal periosteal horizontal mattress sutures and a second layer of continuous locking sutures with 4‐0 PTFE sutures (Cytoplast, Osteogenics Biomedical). Care was taken to confirm complete flap passivity and the absence of membrane exposure.

### 2.6 Membrane Fixation Techniques

- Group 1—No fixation (NF) (Lee et al. 2022): The membrane was passively adapted over the graft without any fixation (Figure 1).
- Group 2—Periosteal sutures (PS) (Neiva et al. 2022): A resorbable 5‐0 polyglycolic acid (PGA) suture (Ethicon Vicryl 5‐0, 18″ Coated Vicryl Braided Absorbable Suture, Ethicon US, Johnson & Johnson, PA, USA) was used to secure the collagen membrane over the grafted site. Following graft placement and membrane adaptation, the suture was passed through the periosteal tissue on the buccal and lingual aspects, approximately 3–5 mm apical to the defect margins, using a curved microsurgical needle. The suture was looped in a horizontal mattress configuration to encircle the membrane and underlying graft material, forming a “Lasso” that gently compressed the membrane against the bone surface, improving graft stability. The suture was tied securely on the buccal side, providing stable fixation without the need for pins or tacks. One suture was placed mesial and one distal to the regenerated area to stabilize the totality of the membrane. Care was taken to avoid perforating the membrane and to maintain full coverage over the grafted area (Figure 1).
- Group 3—Fixation pins (FP) (Urban, Nagursky, et al. 2013): membrane fixation was performed using titanium bone fixation pins (Master pin control kit, Meisinger, USA) to achieve firm stabilization of the collagen membrane over the grafted site. According to Urban, Nagursky, et al.'s (2013) technique, the membrane was placed on top of the alveolar defect and fixed with at least two fixation pins in the lingual aspect, one mesial and one distal. Then, the particulate bone graft was placed into the defect. Lastly, the membrane was tightly stretched and secured by inserting one pin at the mesial and one at the distal border of the buccal aspect of the membrane, approximately 2–3 mm beyond the graft margins. In the present study, only four pins were used to fix the membrane in all cases. Pins were inserted manually using a sterile applicator to ensure perpendicular angulation and full seating against the cortical surface. After verification of membrane immobility and adaptation, any excess membrane was trimmed as needed prior to flap advancement and primary closure (Figure 1).

### 2.7 Cone Beam Computed Tomography (CBCT) Imaging Protocol

All specimens were scanned using a 3D Accuitomo CBCT unit (J. Morita Corp., Kyoto, Japan), a dental imaging system designed for high‐resolution evaluation of craniofacial structures. Scans were performed using the following parameters: 90 kVp, 5 mA, voxel size of 0.30 mm, and a field of view (FOV) of 170 × 120 mm, sufficient to capture the entire grafted region and surrounding bone. Scans for each specimen were taken at three time points: baseline (defect assessment), immediately after membrane placement and fixation (pre‐closure), and following flap closure (post‐closure).

### 2.8 Image Analysis

CBCT Digital Imaging and Communications in Medicine (DICOM) files were imported into BlueSkyPlan software (BlueSkyBio LLC, Libertyville, IL, USA) for image analysis. This software provides volumetric rendering, artificial intelligence (AI)‐driven segmentation, and precise measurement tools tailored for dental and maxillofacial applications. The region of interest (ROI) was defined by the boundaries of the grafted site under the membrane.

**FIGURE 2:**
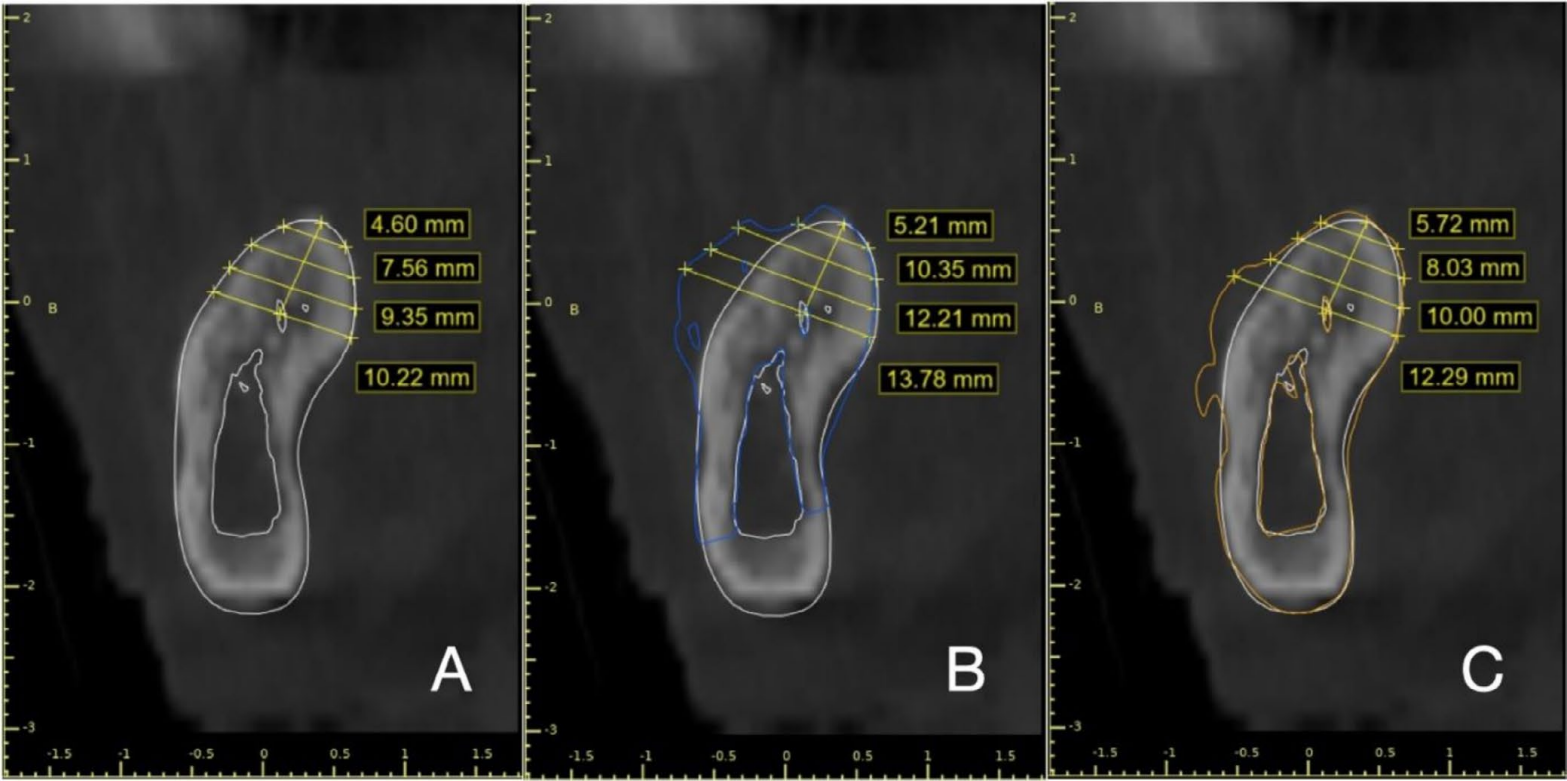
Example of a central linear measurement of the periosteal suture (PS) fixation technique at 1, 3, 5, and 7 mm from the crest. (A) Baseline CBCT. (B) Pre‐closure bone surface aligned to baseline CBCT. (C) Post‐closure bone surface aligned to baseline CBCT.

Linear measurements were performed on standardized cross‐sectional CBCT slices to evaluate graft width at multiple levels. For each grafted site, measurements were taken at the center, 5 mm mesial, and 5 mm distal to the midpoint of the defect. At each of these locations, horizontal graft width was measured at 1, 3, 5, and 7 mm apical to the alveolar crest. Additionally, the baseline ridge width was recorded at the same levels for comparison (Figure 2).

**FIGURE 3:**
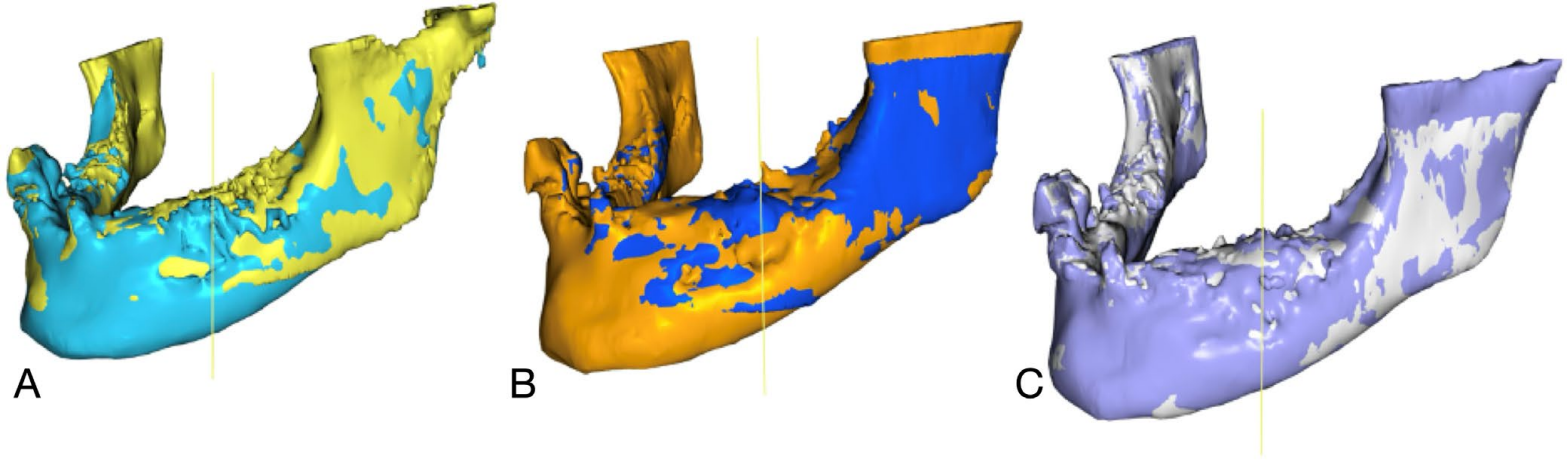
The images show the superimposition of bone surfaces in all three experimental groups compared to baseline. (A) No fixation. (B) Periosteal sutures. (C) Fixation pins.

To ensure consistent measurement positions across all time points (baseline, pre‐closure, and post‐closure) and all fixation techniques, all segmented mandibular volumes were exported as STL files, and the different time points were aligned through spatial references using surface‐based alignment tools in Autodesk Meshmixer (Autodesk Inc., San Rafael, CA, USA). The aligned STL files were then re‐imported into the baseline CBCT volume, allowing all measurements to be performed on identical cross‐sectional slices for each condition, as the baseline CBCT was used for spatial positioning and orientation reference. This workflow ensured accurate and reproducible comparisons of linear graft dimensions across time points and techniques (Figure 3).

Volumetric analysis was conducted using Autodesk Meshmixer (Autodesk Inc., San Rafael, CA, USA) to evaluate the dimensional stability of the graft material before and after flap closure. These STL files were then imported into Meshmixer, where the graft material from each time point (pre‐closure and post‐closure) was isolated and refined to eliminate noise and ensure accurate surface boundaries. The volume of the grafted material at each time point was calculated using Meshmixer's “Analysis > Stability > Volume” tool and recorded in mm^3^.

**FIGURE 4:**
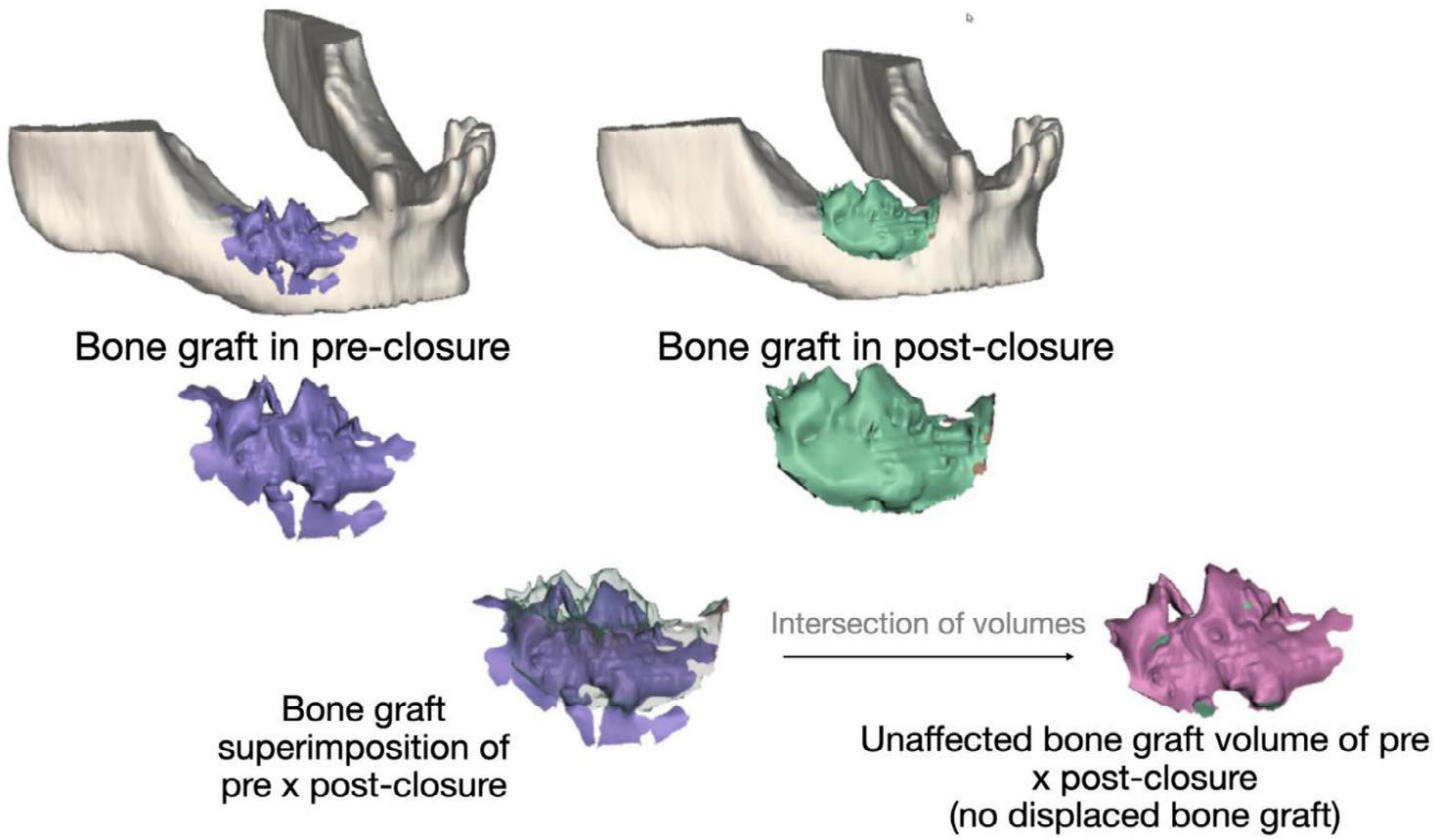
The image sequence shows the process of bone graft volume separation from the mandible and analysis of displacement between pre and post‐closure flap. The above example refers to the Fixation Pin technique and shows minimum volume displacement.

To quantify graft displacement, a Boolean intersection operation was performed between the pre‐ and post‐closure graft volumes. This allowed for the visualization and quantification of overlapping versus displaced graft material. The percentage of displaced volume was then calculated to compare the stability across the three membrane fixation techniques (Figure 4). All measurements were conducted by a single trained and experienced examiner blinded to the experimental groups (N.Z.).

### 2.9 Statistical Analysis

Descriptive statistics were reported as means and standard deviations (SDs) for all continuous variables. The primary outcome—linear dimensional changes of the grafted site—was assessed using a mixed‐effects multilevel multivariable linear regression model. Fixed effects included the baseline ridge width, depth of measurement (1, 3, 5, and 7 mm from the crest), and GBR technique (FP, PS, No Fixation). The specimen identifier was incorporated as a random effect to account for within‐subject correlation across repeated measurements. Two models were constructed:

- Model 1: Adjusted for baseline width and measurement depth.
- Model 2: The model additionally included an interaction term between the GBR technique and measurement depth, aiming to evaluate whether the technique's effect varied with the measurement's vertical position.

Following the regression, Tukey‐adjusted pairwise comparisons were performed on estimated marginal means to assess post hoc differences between GBR techniques.

The secondary outcome, volumetric changes between bone graft and membrane placement versus pre‐ and post‐flap closure, was analyzed using a nonparametric Friedman rank sum test, suitable for paired and non‐normally distributed data with three groups (GBR techniques). Post hoc Wilcoxon signed‐rank tests with false discovery rate (FDR; Benjamini–Hochberg) adjustment were conducted to explore pairwise differences. In addition, within‐group comparisons of pre‐ and post‐flap closure volumes were tested using paired Wilcoxon signed‐rank tests.

For the tertiary outcome, graft stability, a separate variable was defined as the percentage of graft volume that remained stable after flap closure, calculated as follows:

$$\text{Stability}\;\left(\%\right)=\frac{\text{Intersection Volume}}{\mathrm{Pre}‐\text{closure Volume}}\times 100$$

Friedman test followed by FDR‐adjusted Wilcoxon signed‐rank tests were again used to assess differences across GBR techniques.

All analyses were conducted by one investigator with experience in biostatistics (H.S.) using RStudio, with the following packages: tidyverse, lme4, lmerTest, emmeans, rstatix, and ggpubr.

## 3 Results

Linear dimensional changes were assessed across central, mesial, and distal regions from baseline to graft and membrane application and fixation (representing gain) and from pre‐ to post‐flap closure (representing collapse). All groups demonstrated a comparable increase in ridge width after graft placement and membrane placement. The FP technique showed the greatest overall linear gain (2.90 ± 0.84 mm), followed by no fixation (2.81 ± 0.73 mm) and PS fixation (2.67 ± 0.82 mm), with no statistically significant difference between groups in initial bone gain (*p* = 0.618). Site‐specific gains in the central region were highest with the NF (3.34 ± 0.82 mm) and FP (3.26 ± 0.78 mm) techniques. Mesial and distal gains followed a similar trend across groups.

**FIGURE 5:**
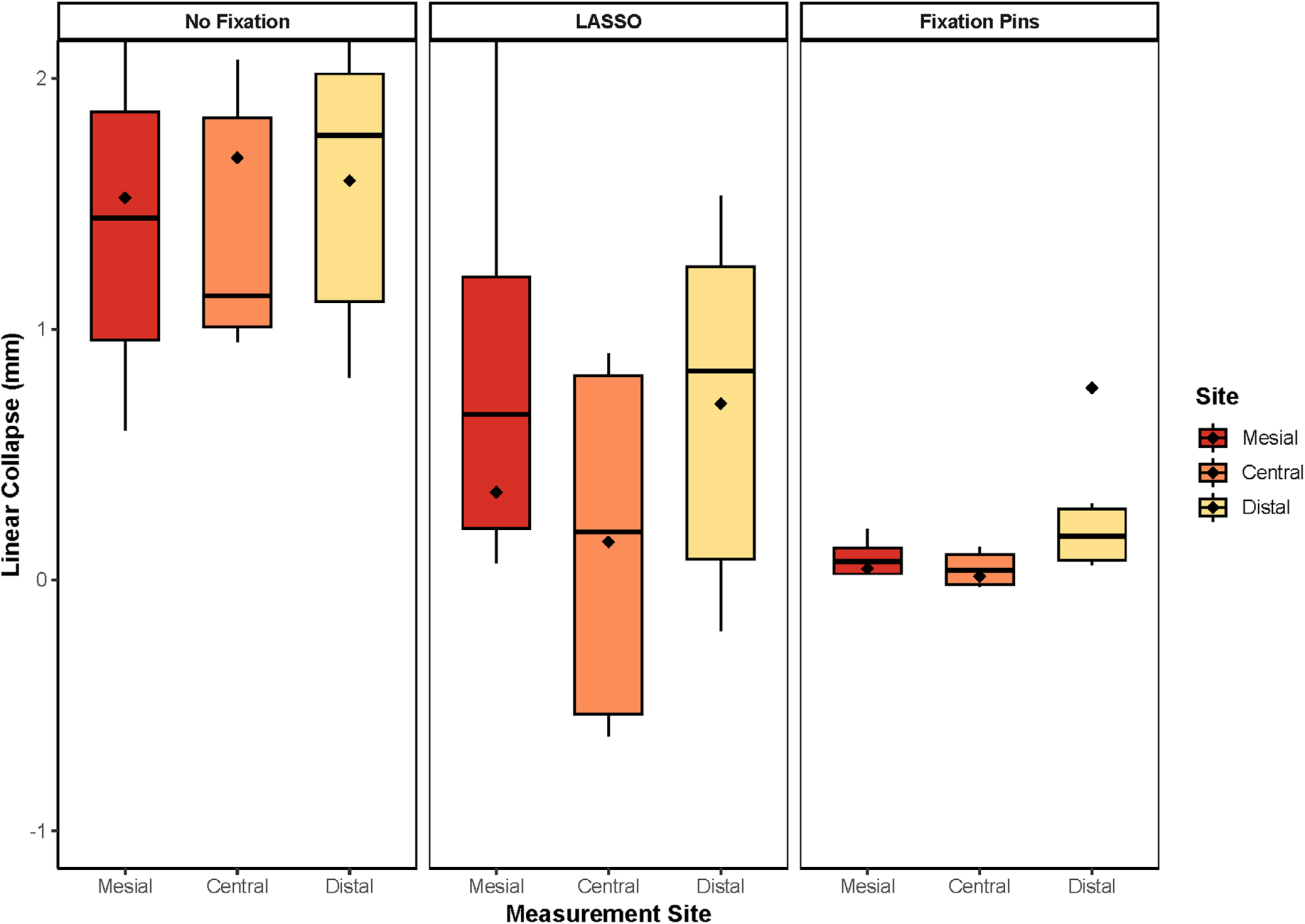
The mean linear width change (in mm) is specified for each group and each measurement site.

**FIGURE 6:**
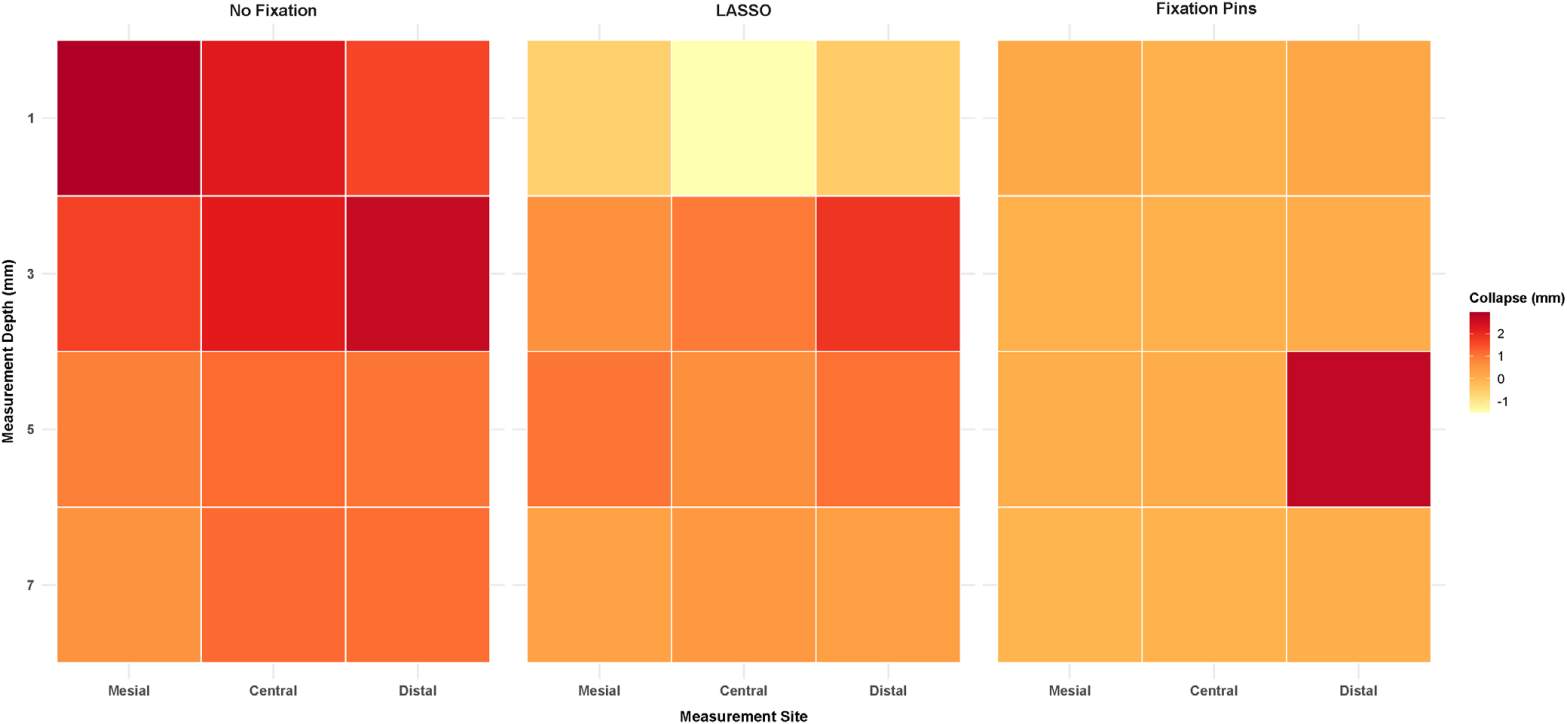
Graft collapse after site closure in each of the study groups and site‐specific assessment (measurement depth and site). Note that the darker color indicates higher collapse.

However, differences became more pronounced when examining collapse following flap closure. The no fixation group exhibited the most substantial collapse across all regions, with a mean collapse of −1.37 ± 0.85 mm, particularly in the central (−1.68 ± 1.13 mm) and distal (−1.59 ± 0.59 mm) areas. In contrast, FP fixation demonstrated minimal collapse (−0.28 ± 0.61 mm), with nearly no reduction observed at the central (−0.01 ± 0.12 mm) and mesial (−0.05 ± 0.15 mm) sites. PS fixation showed intermediate performance (−0.40 ± 0.62 mm mean collapse) (Figure 5). These patterns were consistent across individual sites (Figure 6). For a complete list of site‐specific measurements across depths (1, 3, 5, and 7 mm), see Table 1.

**TABLE 1:** (Top) Mean and standard deviations (SD) of ridge width before application of graft and membrane (Baseline), following membrane placement with respective technique (No Fixation, PS, FP) before closure (Open) and after flap closure (Closed). (Bottom) Mean ridge width changes for all depths combined. This indicates width gain (from graft placement to pre‐closure) and graft collapse (after flap closure compared to pre‐closure).

Timepoint	Measurement point															
	1 mm				3 mm				5 mm				7 mm			
	Central	Mesial	Distal	Mean	Central	Mesial	Distal	Mean	Central	Mesial	Distal	Mean	Central	Mesial	Distal	Mean
**No membrane fixation technique**																
Baseline	6.98 ± 3.2	6.91 ± 3.09	7.64 ± 3.16	7.18 ± 2.96	10.6 ± 3.08	9.82 ± 3.37	10.6 ± 3.41	10.34 ± 3.2	12.25 ± 3.04	11.32 ± 2.88	12.44 ± 3.19	12 ± 3	12.61 ± 2.76	11.81 ± 2.46	13.13 ± 3.4	12.52 ± 2.85
Open	11.39 ± 2.46	11.2 ± 2.82	10.49 ± 3.96	10.57 ± 3.48	14.54 ± 2.94	13.1 ± 2.55	14.45 ± 2.38	14.03 ± 2.5	14.9 ± 2.75	13.21 ± 2.03	15.05 ± 2.64	14.39 ± 2.45	14.98 ± 2.56	12.98 ± 1.97	14.95 ± 3.22	14.3 ± 2.51
Closed	9.16 ± 3.4	8.27 ± 4.21	8.87 ± 3.94	9.22 ± 3.17	12.34 ± 3.13	11.42 ± 3	11.83 ± 3.15	11.86 ± 2.98	13.75 ± 2.96	12.32 ± 2.16	14.02 ± 2.9	13.37 ± 2.63	13.83 ± 2.58	12.38 ± 1.81	13.84 ± 3.43	13.35 ± 2.57
**Periosteal sutures technique**																
Baseline	7.11 ± 3.08	7.78 ± 3.98	7.31 ± 3.92	7.4 ± 3.58	10.34 ± 3.3	10.06 ± 3.47	10.45 ± 3.49	10.28 ± 3.36	11.91 ± 3.03	10.75 ± 2.71	12.3 ± 3.31	11.66 ± 2.88	12.31 ± 2.88	11.9 ± 2.36	12.95 ± 3.26	12.38 ± 2.81
Open	10.4 ± 3.14	10.69 ± 5.07	9.29 ± 4.21	10.13 ± 3.93	14.11 ± 4.15	13.07 ± 3.29	14.2 ± 4.43	13.8 ± 3.88	14.87 ± 3.44	13.83 ± 3.27	14.36 ± 3.61	14.35 ± 3.34	14.5 ± 2.98	13.45 ± 1.77	14.5 ± 3.37	14.15 ± 2.6
Closed	11.89 ± 3.73	11.28 ± 3.63	9.8 ± 5.14	10.99 ± 3.85	13.16 ± 3.64	12.42 ± 2.75	12.33 ± 4.29	12.64 ± 3.41	14.2 ± 2.76	12.8 ± 2.22	13.28 ± 3.65	13.43 ± 2.72	14.02 ± 2.35	13.13 ± 1.55	14.12 ± 2.78	13.76 ± 2.2
**Fixation pins technique**																
Baseline	7.64 ± 3.2	6.5 ± 2.92	7.86 ± 4.21	7.33 ± 3.42	10.7 ± 2.79	9.93 ± 3.34	10.76 ± 3.42	10.46 ± 3.15	12.32 ± 2.86	11.58 ± 3.14	12.81 ± 3.46	12.24 ± 3.1	12.84 ± 2.99	12.15 ± 2.65	13.33 ± 3.75	12.77 ± 3.08
Open	11.66 ± 2.64	10.3 ± 2.22	10.37 ± 4.08	10.78 ± 2.89	14.57 ± 2.17	12.88 ± 2.09	13.89 ± 3.43	13.78 ± 2.47	15.35 ± 2.74	14.24 ± 2.53	15.34 ± 3.1	14.98 ± 2.75	14.96 ± 3.13	14.2 ± 2.4	15.43 ± 3.28	14.86 ± 2.9
Closed	11.67 ± 2.52	10.15 ± 2.42	10.15 ± 3.94	10.66 ± 2.9	14.54 ± 2.19	12.86 ± 2.07	13.81 ± 3.39	13.74 ± 2.47	15.28 ± 2.69	14.17 ± 2.53	12.65 ± 6.41	14.03 ± 3.01	15 ± 2.95	14.26 ± 2.45	15.35 ± 3.3	14.87 ± 2.87
	**Changes between timepoints**															
	**Baseline to open flap (linear gain)**								**Open flap to flap closure (linear collapse)**							
	**Central**		**Mesial**		**Distal**		**Mean gain (*p*)**		**Central**		**Mesial**		**Distal**		**Mean collapse**	
No membrane fixation	3.34 ± 0.82		2.65 ± 1.11		2.78 ± 0.78		2.81 ± 0.73 (0.011)		−1.68 ± 1.13		−1.52 ± 0.82		−1.59 ± 0.59		−1.37 ± 0.85	
Periosteal sutures	3.05 ± 0.99		2.64 ± 0.74		2.33 ± 1.13		2.67 ± 0.82 (0.002)		−0.15 ± 0.75		−0.35 ± 1.82		−0.7 ± 0.73		−0.4 ± 0.62	
Fixation pins	3.26 ± 0.78		2.87 ± 1.2		2.57 ± 0.79		2.9 ± 0.84 (0.003)		−0.01 ± 0.12		−0.05 ± 0.15		−0.77 ± 1.72		−0.28 ± 0.61	

### 3.1 Mixed‐Effects Regression Results on Outcome of Linear Changes

In the primary regression analysis (Model 1), which adjusted for baseline ridge width and depth of measurement, both GBR techniques were associated with significantly less graft collapse compared to the no‐fixation group. Specifically, the PS group showed 0.96 mm (SE = 0.37, *p* = 0.012), and the FP group showed 1.11 mm (SE = 0.37, *p* = 0.004) less reduction when the no fixation group served as the reference. Neither baseline width (*p* = 0.212) nor depth of measurement (*p* = 0.729) was significantly associated with the amount of collapse (Table 2).

**TABLE 2:** Mixed‐effects multilevel multivariate linear regression analysis on outcome of ridge width change from pre‐closure to post‐closure timepoints and post hoc Tukey's honest significant difference (HSD) test for pairwise comparisons. Note that two models were constructed: The first adjusted for baseline ridge width and measurement depth, and the second included interaction terms between GBR technique and depth of measurement.

Model 1: Adjusted for depth and baseline		
Variable	Estimate (SE)	*p*
Intercept	0.79 (0.53)	0.14
Technique (reference: no fixation)		
PS	−0.96 (0.37)	0.012
FP	−1.11 (0.37)	0.004
Baseline ridge width (mm)	0.07 (0.05)	0.212
Depth of measurement (mm)	−0.03 (0.08)	0.729

Model 2: Interaction model GBR technique × depth		
Variable	Estimate (SE)	*p*
Intercept	1.36 (0.65)	0.04
Technique (reference: no fixation)		
PS	−2.15 (0.76)	0.006
FP	−1.67 (0.76)	0.032
Depth of measurement (mm)	−0.18 (0.13)	0.168
Baseline ridge width (mm)	0.07 (0.05)	0.194
PS × Depth	0.30 (0.17)	0.079 †
FP × Depth	0.14 (0.17)	0.405

Tukey's honest significant difference (HSD) test (adjusted pairwise comparisons based on model 2)		
Contrast	Difference (SE)	*p*
NF − PS	0.96 (0.37)	0.031
NF − FP	1.11 (0.37)	0.011
PS − FP	0.15 (0.37)	0.92

In the interaction model (Model 2), which included a GBR‐by‐depth interaction term, the protective effects of both GBR techniques on the outcome of width collapse remained significant. Compared to the no fixation group, PS was associated with 2.15 mm (SE = 0.76, *p* = 0.006) and FP with 1.67 mm (SE = 0.76, *p* = 0.032) less collapse. The interaction between PS and depth trended toward significance (*p* = 0.079), suggesting a possible depth‐dependent effect. When the interaction between GBR technique and depth was introduced (Model 2), the estimated protective effect of the PS technique became more pronounced, shifting from −0.96 mm (SE = 0.37) in Model 1 to −2.15 mm (SE = 0.76), while the estimate for FP shifted from −1.11 mm (SE = 0.37) to −1.67 mm (SE = 0.76). This suggests that the performance of PS, in particular, may vary depending on the vertical level of the measurement, as reflected by the near‐significant interaction term (*p* = 0.079) (Table 2).

#### 3.1.1 Post Hoc Comparisons

Tukey‐adjusted pairwise comparisons (based on Model 2) confirmed significant differences between GBR techniques. The no fixation group had significantly greater collapse compared to both PS (mean difference = 0.96 mm, *p* = 0.031) and FP (mean difference = 1.11 mm, *p* = 0.011). However, the difference between PS and FP was not statistically significant (mean difference = 0.15 mm, *p* = 0.92) (Table 2).

### 3.2 Volumetric Changes and Graft Stability

In the volumetric analysis, the no fixation group showed the greatest mean volume reduction from pre‐ to post‐flap closure (−247.05 ± 149.51 mm^3^), followed by PS (−95.97 ± 165.08 mm^3^). The FP group showed the smallest, and only subtle volume reduction (−6.69 ± 87.40 mm^3^). Within‐group comparisons using paired Wilcoxon signed‐rank tests revealed a statistically significant reduction in volume for the No fixation group (*p* = 0.031), while no significant reduction was observed for PS (*p* = 0.313) or FP (*p* = 0.688) techniques. Moreover, between‐group comparison using Friedman's test revealed a significant difference in volumetric change across GBR techniques (*p* = 0.030). Post hoc pairwise comparisons (FDR‐adjusted) showed no significant difference between No Fixation and PS (*p* = 0.313), a trend toward significance between No fixation and FP (*p* = 0.094), and no significant difference between PS and FP (*p* = 0.313) (Table 3).

**TABLE 3:** Volumetric outcomes' comparison among the three groups (top). Volumetric changes from application of graft and membrane versus flap closure and respective statistical models (bottom). Comparison of the outcome of graft stability percentage (%) and intergroup comparisons.

Volumetric changes				
GBR technique	Open flap to closed flap volume change (mean ± SD, mm^3^)	Wilcoxon paired *p* value (intragroup)	Friedman test (intergroup)	Post hoc (FDR‐adjusted *p* value)
NF	−247.05 ± 149.51	0.031		NF vs. PS: 0.313
PS	−95.97 ± 165.08	0.313	*p* = 0.030	NF vs. FP: 0.094
FP	−6.69 ± 87.40	0.688		PS vs. FP: 0.313

Volumetric graft stability (%)			
GBR technique	Stability (mean ± SD, %)	Pairwise comparison (FDR‐adjusted *p* value)	Friedman test (intergroup)
NF	44.4% ± 17.9%	PS vs. FP: 0.031	*p* = 0.002
PS	70.1% ± 4.5%	PS vs. NF: 0.031	
FP	81.2% ± 8.2%	FP vs. NF: 0.031	

Graft stability, defined as the percentage of graft volume that remained stable from open to closed flap, was highest in the FP group (81.2% ± 8.2%), followed by PS (70.1% ± 4.5%) and no fixation (44.4% ± 17.9%). The Friedman test again showed significant intergroup differences in graft stability (*p* = 0.002). All post hoc comparisons (FP vs. No Fixation, PS vs. No Fixation, and PS vs. FP) were statistically significant after FDR correction (*p* = 0.031 for all comparisons) (Table 3).

## 4 Discussion

This study demonstrated that both fixation techniques—periosteal sutures and titanium pins—effectively minimized graft collapse, with fixation pins consistently showing superior performance across linear, volumetric, and stability outcomes. While periosteal sutures offered moderate improvement compared to no fixation, the use of fixation pins resulted in minimal displacement of the graft material and the highest percentage of graft retention after flap closure. Overall, fixation, particularly with pins, was proven to be critical for maintaining the integrity of the grafted site. Our results indicate that the no‐fixation group experienced the most substantial collapse across all regions, with an average collapse of −1.37 ± 0.85 mm. This was particularly evident in the central region (−1.68 ± 1.13 mm). Given these findings, the present data suggest that the use of collagen membranes without fixation in non‐contained defects may be associated with increased immediate graft displacement following flap closure. However, the clinical relevance of this displacement for long‐term ridge reconstruction and future implant placement remains multifactorial and should be interpreted in light of defect characteristics, surgical technique, and patient‐related factors, as well as the inherent limitations of this ex vivo model.

Taken together, the head‐to‐head Tukey contrasts and the volumetric data demonstrated a more coherent clinical picture. In linear terms, both fixation approaches limited ridge‐width collapse relative to no fixation. FP and PS did not differ statistically from each other (Δ = 0.15 mm, *p* = 0.92) in this particular analysis. However, when volumetric behavior is considered, FP clearly outperformed PS: graft stability averaged 81% with FP versus 70% with PS and 44% with no fixation, and FP showed almost negligible volume loss (−7 mm^3^) compared with PS (−96 mm^3^) and no fixation (−247 mm^3^). Although some individual comparisons did not reach conventional significance thresholds—likely a function of sample size—the convergence of linear, volumetric, and stability metrics indicates a clinically relevant order, that is, fixation pins provide the most predictable dimensional maintenance, periosteal sutures offer an intermediate benefit, and omitting fixation leaves the graft highly vulnerable to collapse. A depth‐by‐technique interaction further accentuates the difference between the two fixation methods: the PS technique showed an almost‐significant positive slope (≈0.30 mm extra collapse for every millimeter deeper measurement), suggesting its protective effect diminishes as the assessment moves apically, whereas the FP (pin) technique exhibited no depth dependence, maintaining its stability throughout the augmentation area.

The influence of flap closure on graft displacement observed in the present study is consistent with previous preclinical evidence. In an in vitro model of one‐wall horizontal defects, Mertens et al. (2019) demonstrated that primary wound closure alone can induce significant displacement of particulate graft materials, particularly in non‐contained defects located outside the bony envelope, and that the use of stabilization strategies substantially reduces this effect. Their findings further highlighted that graft displacement is most pronounced in the coronal portion of the augmented site, underscoring the importance of mechanical stabilization during early wound closure (Mertens et al. 2019).

Recent literature reviews have emphasized the critical role of defect morphology, specifically whether the defect is contained within or extends outside the bony housing, in determining the expected outcomes of horizontal ridge augmentation (Buser et al. 2023; Yu et al. 2023). This concept is further supported by clinical evidence from the Quirynen group, who demonstrated that non‐contained defects are associated with reduced predictability and increased need for space‐maintaining strategies during regenerative procedures (Quirynen et al. 2023). Recent clinical studies have demonstrated that GBR leads to an average bone gain ranging from 2.5 to 4.0 mm as measured 1 mm from the top of the implant platform/crest of the ridge (Lorenz et al. 2025; Jung et al. 2025). Even with successful outcomes, healing complications can sometimes arise: membrane exposure is a frequent occurrence, with an average incidence reported between 11% and 23% (Wessing et al. 2018; Tay et al. 2022). Consistent with previous clinical and preclinical evidence, the use of fixation devices such as titanium pins or miniscrews provides effective stabilization of absorbable membranes during guided bone regeneration (Urban, Lozada, et al. 2013; Urban, Nagursky, et al. 2013; Siar et al. 2011; Carpio et al. 2000; Lorenzoni et al. 2002). In the present study, only four pins were used to fix the membrane in all cases, with more pins likely providing even further stabilization, mostly in longer atrophic sites.

One alternative to fixation pins is the periosteal vertical mattress sutures technique (Urban et al. 2016). In this technique, two vertical mattress sutures were used to stabilize the membrane. The sutures were threaded through the apical periosteum and the lingual periosteum, with one suture on either side of the graft site. A recent pilot study examined the increase in bone thickness at 6 months from staged GBR procedures using periosteal mattress sutures. The authors reported a significant increase in bone thickness, with an average of 3.4 ± 1.3 mm, and noted that wound healing was uneventful in all cases (Moreno et al. 2023). The findings of our study indicate that the PS technique had a mean collapse of −0.40 ± 0.62 mm. The main advantage of this fixation technique is that it is a “metal‐free” approach, reducing time, costs, risks, and discomfort for the patient. Despite these benefits, our study positions PS in a middle tier of effectiveness, while the best results were obtained using FP. This FP technique demonstrated minimal collapse (−0.28 ± 0.61 mm), with virtually no reduction at the central augmentation area (−0.01 ± 0.12 mm).

Another alternative to titanium pins is resorbable magnesium pins, which are biocompatible and demonstrate mechanical strength comparable to titanium, while offering the added advantage of being fully resorbable, thereby eliminating the need for a secondary removal surgery (Franke and Korzinskas 2024; Windhagen et al. 2013). Furthermore, magnesium generates fewer imaging artifacts in radiographic and cross‐sectional imaging modalities than titanium, enhancing postoperative assessment (Steigmann et al. 2020). Recent experimental and clinical data support the use of magnesium‐based screws and membranes in regenerative procedures, demonstrating successful outcomes in terms of new bone formation and implant stability (Steigmann et al. 2020).

Beyond fixation pins and sutures, recent experimental research has explored alternative device‐based approaches to enhance graft stability during guided bone regeneration. Bienz et al. (2025) introduced a novel bioresorbable, implant‐supported device designed to stabilize the augmented volume during flap closure without the use of fixation pins. In an in vitro CBCT study on pig mandibles, the device achieved horizontal augmentation thickness comparable to an L‐shaped DBBM‐C construct stabilized with titanium pins, while significantly reducing procedural time and technical difficulty. Although evaluated in a peri‐implant dehiscence model, this concept supports the rationale that mechanical stabilization at the time of wound closure rather than fixation pins alone plays a central role in maintaining graft stability (Bienz et al. 2025).

A significant gap in the literature relates to the volumetric changes occurring before and after flap closure; this was a primary outcome of our investigation. We expressed graft stability as the percentage of graft volume that remained stable after flap closure. The highest stability was observed in the FP group (81.2% ± 8.2%), followed by PS (70.1% ± 4.5%) and No fixation (44.4% ± 17.9%). Notably, we found statistically significant differences both within and between these groups. We detected a true volumetric difference driven by the only group with a significant intragroup loss (NF), yet our small sample size muted most intergroup *p* values. Importantly, the graft‐stability metric, which was fully significant for every pairwise comparison, confirms the clinical hierarchy and superiority of FP followed by PS followed by NF.

It should also be highlighted that the FP group exhibited the least volume reduction (−6.69 ± 87.40 mm^3^), which was supported by inter‐ and intragroup statistical analysis, making it the most predictable technique for avoiding graft material displacement and loss of bone graft volume. These results closely align with findings from a recent ex vivo study on pig hemimandibles (Raabe, Cafferata, Couso‐Queiruga, et al. 2025), which reported a difference in graft material thickness (GMT) before and after primary wound closure of −35.1% ± 14.9% for the modified periosteal‐released incision (MPRI‐PS) and −31.4% ± 18.8% for the mucosal detachment technique (periosteal pouch flap technique) with a periosteal suture (Raabe, Cafferata, Couso‐Queiruga, et al. 2025).

In comparison, the results observed in this cadaver study seem less encouraging. This discrepancy may arise from differences in flap advancement techniques studied in the pig model, which involved a periosteal pouch that might not provide enough tissue advancement. However, the authors of that study noted that this difference was not significant between the groups, suggesting that the flap advancement technique does not affect graft material displacement during primary wound closure (Raabe, Cafferata, Couso‐Queiruga, et al. 2025). In the present study, we employed the same flap design across all groups to minimize variability and maintain focus on the fixation method and its effect on volume stability.

Multiple limitations need to be acknowledged in this study. One of the more obvious limitations of our work pertains to the study design itself: the ex vivo design does not account for confounding factors that could affect graft material displacement, such as postoperative swelling, hematoma formation, mucosal movements during mastication or facial expressions, and physical contact by the patient. As such, the outcomes observed may not be fully representative of those seen in actual clinical scenarios involving live functioning patients, since the procedures were done in edentulous sites where a true indication for horizontal guided bone regeneration based on a prosthetically driven implant placement was not evaluated. For the PS technique, other factors to consider that might have some influence on its predictability in clinical situations, which could not be tested in the present model, include the tensile strength and resorption rate of the sutures and the biodegradation timeline of the absorbable suture material. Therefore, the durability of the stabilizing effect of periosteal sutures remains an area that requires more research. Furthermore, although the flap release was performed before the regenerative procedures to standardize the tensile strength of the flaps, the three procedures were performed in a non‐randomized sequential order in all the defects, which could have played a role on the consecutive tensile strength of the flaps. Lastly, although examiner blinding was attempted, the radiopacity of the fixation pins made the DICOM files pertaining to the FP group easily identifiable, limiting the blinding.

Moreover, our findings do not account for long‐term biological processes such as tissue remodeling or graft contraction over time. Because this ex vivo model did not evaluate healing, vascularization, membrane degradation, or new bone formation, the findings should be interpreted as evidence of immediate mechanical stability rather than clinical regenerative superiority. These limitations should be considered when interpreting the translational relevance of our results, and numerical data reported here should therefore be interpreted with caution. Future research could explore the effects of using fibrin sealant, self‐contained (putty‐like) graft material, or a combination with platelet‐rich fibrin (PRF) or its derivatives on the displacement during primary wound closure, as well as the implications of a tension‐free flap design and the additional use of connective tissue grafts.

## 5 Conclusion

Within the limitations of this ex vivo study, membrane fixation significantly influenced graft stability in GBR. Fixation pins offered the highest dimensional and volumetric stability, while the periosteal fixation sutures method offered a promising, clinically relevant, metal‐free alternative with acceptable performance. Collagen membranes without fixation in non‐contained defects are not suggested.

## Author Contributions

**Muhammad H. A. Saleh:** conceptualization, investigation, resources, writing – review and editing. **Javier Calatrava:** conceptualization, project administration, writing – review and editing. **Nataly Zambrana:** data curation, visualization, writing – review and editing. **Lucrezia Parma‐Benfenati:** writing – original draft, writing – review and editing. **Hamoun Sabri:** formal analysis, visualization, writing – review and editing. **Istvan Urban:** writing – review and editing. **Hom‐Lay Wang:** writing – review and editing.

## Funding

The authors have nothing to report.

## Conflicts of Interest

The authors declare no conflicts of interest.

## Data Availability

The data that support the findings of this study are available on request from the corresponding author. The data are not publicly available due to privacy or ethical restrictions.
